# Mouse Models of Mineral Bone Disorders Associated with Chronic Kidney Disease

**DOI:** 10.3390/ijms24065325

**Published:** 2023-03-10

**Authors:** Ariane Zaloszyc, Julie Bernardor, Justine Bacchetta, Gilles Laverny, Claus Peter Schmitt

**Affiliations:** 1 Service de Pédiatrie 1, Hôpital de Hautepierre, Hôpitaux Universitaires de Strasbourg, 67000 Strasbourg, France; 2 Institut de Chimie et Procédés pour l’Energie, l’Environnement et la Santé (ICPEES), UMR-7515 CNRS-Université de Strasbourg, 67087 Strasbourg, France; 3 Service de Néphrologie Pédiatrique, CHU de Nice, Hôpital Archet, 06202 Nice, France; 4 Faculté de Médecine, Université Côte d’Azur, 06107 Nice, France; 5 INSERM UMR S1033 Research Unit, 69008 Lyon, France; 6 Reference Center for Rare Renal Diseases, Pediatric Nephrology Rheumatology and Dermatology Unit, Hopital Femme Mère Enfant, 69500 Bron, France; 7 Reference Center for Rare Diseases of Calcium and Phosphate Metabolism, Pediatric Nephrology Rheumatology and Dermatology Unit, Hopital Femme Mère Enfant, 69500 Bron, France; 8 Lyon Est Medical School, Université Claude Bernard Lyon 1, 69003 Lyon, France; 9 Institut de Génétique et de Biologie Moléculaire et Cellulaire, 67400 Illkirch, France; 10 Centre National de la Recherche Scientifique, UMR7104, 67400 Illkirch, France; 11 Institut National de la Santé et de la Recherche Médicale, U1258, 67400 Illkirch, France; 12 IGBMC, Université de Strasbourg, 67400 Illkirch, France; 13 OSCAR, French Network for Rare Bone Diseases, 94270 Le Kremlin-Bicêtre, France; 14 Center for Pediatric and Adolescent Medicine, University Hospital Heidelberg, 69120 Heidelberg, Germany

**Keywords:** CKD, mice, CKD–MBD, renal osteodystrophy

## Abstract

Patients with chronic kidney disease (CKD) inevitably develop mineral and bone disorders (CKD–MBD), which negatively impact their survival and quality of life. For a better understanding of underlying pathophysiology and identification of novel therapeutic approaches, mouse models are essential. CKD can be induced by surgical reduction of a functional kidney mass, by nephrotoxic compounds and by genetic engineering specifically interfering with kidney development. These models develop a large range of bone diseases, recapitulating different types of human CKD–MBD and associated sequelae, including vascular calcifications. Bones are usually studied by quantitative histomorphometry, immunohistochemistry and micro-CT, but alternative strategies have emerged, such as longitudinal in vivo osteoblast activity quantification by tracer scintigraphy. The results gained from the CKD–MBD mouse models are consistent with clinical observations and have provided significant knowledge on specific pathomechanisms, bone properties and potential novel therapeutic strategies. This review discusses available mouse models to study bone disease in CKD.

## 1. Introduction and Rational for Mouse Models of CKD–MBD

Chronic kidney disease (CKD) is highly prevalent and induces mineral and bone metabolism disorders (MBD), even in the early stages of CKD [1,2]. CKD–MBD encompasses abnormalities of calcium and phosphate metabolism induced by the deregulation of parathyroid hormone (PTH), fibroblast growth factor 23 (FGF23) and vitamin D homeostasis that lead to renal osteodystrophy (ROD), a term used to describe the different patterns of skeletal abnormalities in patients with CKD and extra-osseous calcifications. The latter comprises the rare but life-threatening soft tissue calcification of the skin (namely calciphylaxis), as well as the highly prevalent vascular calcifications, essentially contributing to an exceedingly high cardiovascular morbi-mortality [2,3,4,5,6,7,8].

Patients with CKD present a broad spectrum of bone histological changes that include mineralization and bone turnover abnormalities that reduce bone mass and strength; in contrast, bone volume is usually preserved [9,10,11,12]. Histological bone alterations are driven by the stage of CKD and the associated abnormal hormone and mineral metabolism, as well as by the therapeutic interventions, with mineralization abnormalities being more prevalent in children than in adults [11]. Thus, the type of bone disorder evolves over time, and the disease pattern may change substantially, e.g., from a PTH-induced high turnover bone disease to suppression of PTH by high dose active vitamin D treatment and/or high calcium intakes, resulting in the histological phenotype of an adynamic bone, which confers a particularly high risk of vascular calcification since calcium binding capacity of the bone is suppressed [13].

The pathophysiology of CKD–MBD is complex and still not fully understood; studies for the in-depth pathophysiological understanding of disease development and progression and for improved clinical management are urgently needed. However, randomized controlled trials in the field of CKD–MBD are costly and challenging due to the high heterogeneity of bone disorders, the variety of treatments with limited standardization, the underlying diseases and associated treatments affecting bone health per se, and the fact that bone evaluation is not well standardized [14]. Metabolic diseases such as oxalosis or cystinosis have a direct toxic effect on bone [15,16]. Moreover, clinical studies, at best, require repeated bone biopsies but are hardly performed in clinical routines due to their invasiveness [17].

Thus, preclinical animal models represent a valuable tool to overcome these major barriers and limitations. In a controlled environment with standardized conditions of CKD and mineral supply, animal models help to identify specific underlying molecular mechanisms, characterize the role of various contributing factors and determine the efficiency of specific treatments of CKD–MBD. Several CKD–MBD mouse models have been developed that exhibit vascular calcifications [18]. The present review gives an overview of these models and discusses their contribution to the understanding of ROD in CKD–MBD.

## 2. The Mouse Model to Mimic Human CKD–MBD

In 1987, Gagnon et al. were the first to establish stable CKD conditions in mice by nephrectomy and contralateral kidney electrocauterization, which resulted in similar biological and bone alterations to those observed in humans with CKD–MBD [19]. Mice and humans develop similar hormone dysregulation, and toxin accumulation, usually characterized by increased serum creatinine and urea concentrations, hyperphosphatemia and increased circulating PTH and FGF23 levels. In addition, some mouse models develop vascular calcifications and display a large spectrum of bone abnormalities encountered in humans. A bone/vascular axis has been described in two surgery-induced CKD mouse models with high bone turnover, i.e., a relationship between aortic mineral elements and calcifications and the type of osteodystrophy [20,21]. 

Since the first description, many histological and/or micro-CT bone analyses have been performed in mice with CKD. The objectives of these studies were diverse, including the characterization of CKD-associated bone disease, the relationship between bone and vascular disease, and the impact of existing or new therapies. The phenotype and severity of ROD are highly variable and depend on age, gender, the genetic background of the mice, the degree of CKD and secondary hyperparathyroidism (SHPT) achieved and the dietary regime (and notably the calcium and phosphate contents). In addition, the time interval between CKD induction and bone analysis is critical. Moreover, there is a lack of consistency in the parameters assessed, especially by histology and micro-CT.

Other species to study CKD–MBD were mainly rats, applying a 5/6 nephrectomy or adenine diet. This, however, precludes genetic modifications; only spontaneous development of autosomal dominant polycystic kidney disease could be used in rats to study CKD–MBD [18,22]. Other animal models include dogs, cats and rabbits. Substantial species-specific variations have to be considered, e.g., in vitamin D metabolism. Rabbit kidneys are resistant to adenine toxicity [23]. CKD-related osteodystrophy hardly develops in cats [24].

## 3. Mouse Models of CKD–MBD 

### 3.1. Induction of CKD

Since this first description of a CKD-mouse model, several methods of CKD induction have been used. Here, we summarize the various models used to study ROD, as extensive reviews on CKD in mice were previously performed [25,26].

Four main categories of CKD–MBD mouse models were identified, namely CKD induced by surgery, nephrotoxic compounds, spontaneous CKD and genetic engineering [26]; they are summarized in Table 1. In these models, the age of CKD induction was highly variable, ranging from 5 to 38 weeks. CKD degree was progressively increasing over time, mainly in genetic models of CKD. Surgical models were the most frequently used [19,20,21,27,28,29,30,31,32,33,34,35,36,37,38,39,40,41,42,43,44,45,46,47,48], as they offered the possibility to compare the phenotype before and after surgery and were described as stable over time. Subtotal nephrectomy, so-called 5/6th nephrectomy, mimics quite well the process of reduction in nephron mass occurring in humans during renal failure. The surgical models followed the original description from Gagnon et al. [19], with a unilateral nephrectomy and an electrocauterization of the remnant kidney to reduce the total renal mass to 1/6th [21,28,29,30,31,32,37,38,40,41,42,43,44,46,47,48,49,50,51]. The degree of cauterization is critical as the amount of remnant kidney tissue defines the CKD severity [30]. Alternative surgical methods following the unilateral nephrectomy were polar excision of the remnant kidney [20,27,33,34,35,36,39] or ligation of branches of the renal artery [45]. The surgery was mostly done in two steps with a time interval of one to two weeks but was occasionally performed in one step [36,45]. However, 5/6th nephrectomy is challenging in mouse, due to the small size and the low blood volume, and may lead to a high mortality rate. Consequently, other models were developed.

Adenine feeding was first used in rats and leads to CKD due to the crystallization of adenine metabolites in proximal tubules [25,69]. The major advantage of the adenine model is the avoidance of surgery and its associated complications. The protocol was then adapted to mice but required a casein-containing diet for the appetence. In CKD–MBD studies in mice, a 0.2% adenine diet was used for 2 [64], 5 [65], 6 [52,66,67], 8 [46], 12 weeks [53] or between 6 and 12 weeks [54]. 

Frausher et al. reported that Brown agouti/2 (DBA/2) mice fed with a high phosphate diet develop progressive calcifications and CKD [55]. Some authors used both groups of mice with surgically induced CKD and adenine-induced CKD [46,64], with the degree of CKD being higher in the latter in one study [46].

Genetically engineered mouse models comprise the Juvenile Cystic Kidneys (JCK) mice (a model of human Autosomal Dominant Polycystic Kidney Disease) [57,58], the Col4a3^−/−^ and Col4a5^y/−^ mice (models of Alport disease) [59,60,61,62] and the iCTCFpod^−/−^ mice (a model of nephrotic syndrome with kidney failure) [63]. LDLR^−/−^ mice fed a high-fat diet are prone to develop the metabolic disease, characterized by insulin resistance, type 2 diabetes and atherosclerosis, and can be considered as an early CKD model. This model was generally used with complementary surgery to increase the degree of CKD [29,30,31,41]. ApoE^−/−^ mice develop hypercholesterolemia and atherosclerosis, and following surgical kidney mass reduction, were used as a combined CKD- and hypercholesterolemia-induced vascular disease model, i.e., a model of advanced metabolic syndrome [21,42,48]. 

Genetically engineered mice were also used to generate CKD or to explore pathways in the CKD–MBD pathogenesis [21,27,28,30,31,36,42,43,44,57,58,59,60,61,63,64,65,66,67]. 

### 3.2. CKD Mouse Models Combined with High- or Low-Phosphate Diet

Phosphate-enriched diets (HP diet) have been used to enhance the severity of SHPT in many CKD–MBD mouse studies [20,27,34,35,36,37,38,44,53,54,55,56,57,58,64], see Table 1. An increase in vascular disease has been observed in mice with CKD and HP diet [18], accompanied by a similar increase in bone alterations [37,38,44]. The recommended minimal standard dietary phosphate content is 0.3% phosphate together with 0.5% calcium [70], while in CKD–MBD studies, diets usually contained 0.5 to 0.9% phosphate per kg diet [20,21,27,28,29,34,35,36,37,38,40,42,44,48,52,53,54,55,56,57,58,59,64,65,68] or even up to 1.2% [67]. Phosphate-enriched diets contained 0.9 to 2% of phosphate [20,27,34,35,36,37,38,44,53,54,55,56,57,58,64]. These diets were administered over periods ranging from 4 days to 18 weeks [20,27,34,35,36,37,38,44,53,54,55,56,57,58,64]. Interestingly, an HP diet itself can lead to bone structure changes [38,44] and to HPT, which is amplified by CKD [44]. Some authors used a combination of HP diets together with a high calcium diet of 2% calcium [27,34] and found restoration of bone mass [34]. 

To demonstrate specific CKD-induced effects on bone independent of the dysregulation of PTH, active vitamin D, calcium and phosphate, Lund et al. applied a low phosphate diet (0.2%) in CKD mice and supplemented calcitriol to compensate for the lack of renal synthesis of the bioactive form of vitamin D and to prevent HPT and by this was able to also maintain serum phosphate and calcium levels in the normal range [40]. These mice developed very low turnover bone, i.e., an adynamic bone disease with depressions in osteoblast number, bone formation and mineral apposition rate. Zhang et al. used 0.2% and 0.02% phosphate diets in Alport mice to study the relation between phosphate supply and FGF23. Fractional excretion of phosphate was independent of serum FGF23 levels [62].

### 3.3. Impact of Strain, Gender, Age

The susceptibility to CKD and to renal osteodystrophy depends on the mouse strain and gender. The C57BL/6 background has been used most frequently but is relatively resistant to the development of glomerulosclerosis, proteinuria and hypertension [18,25,26]. DBA/2 [37,38] and Crlj:CD1 mice [35] develop CKD–MBD, with a full spectrum of high turnover ROD in mice fed with an HP diet. 

Bone analyses were performed after highly variable time intervals following the induction of renal injury, ranging from 3 to 20 weeks [20,32,35]. CKD duration impacts the bone, with longer CKD duration substantially worsening bone disease [35,58]. Kadokawa et al. described an age-related regression of the trabecular architecture that is accelerated by CKD [35].

The studies were conducted with variable numbers of animals per group, with a minimal number of only 3 to 4, and were performed in males [20,27,32,33,34,35,39,40,43,44,45,53,54,57,60,61,65,67,68], in females [19,21,28,36,38,42,47,48,49,50,51] or used interchangeably in females and males [29,41,46,52,59,62,63,66]. Surprisingly no gender impact was taken into consideration in most trials. A slightly more severe bone phenotype was reported in males than in females in C57BL/6 mice treated with adenine [52,66].

## 4. Analysis of CKD–MBD Mouse Model

### 4.1. Biochemical Parameters: Assessment of CKD

The degree of renal impairment in CKD mice was mostly estimated by the increase in urea (blood urea nitrogen; BUN) [19,20,21,28,29,30,31,32,33,35,36,37,38,39,40,41,42,43,44,46,47,48,49,50,51,53,54,55,56,57,58,59,61,63,64,65,66,67]. While in some models, BUN levels were similar to control [30,31,43], in others, it was 6- to 8-fold increased [33,58,61,63,64]. In some studies, renal function was also estimated by serum creatinine measurements using enzymatic or colorimetric assays, which mostly yielded increases of 1.3- to 9-fold compared to controls but exceptionally up to 40-fold [19,20,27,28,33,39,44,53,54,56,58,59,62,63,65,68]. Alternatively, the 24 h-creatinine clearance was evaluated, and a decrease of 2.6- to 7.3-fold was observed in CKD mice [53,54]. More complex methods of estimating the glomerular filtration rate (GFR), such as inulin clearance, showed reductions from 30 to 85% compared to control mice [30,31,43,55,60]. Importantly, CKD severity was difficult to address due to the important heterogeneity within a given group in some studies, as well as the inter-study variability.

In recent years, new methods to assess GFR have been developed in laboratory animals, and their principle strengths and limits have already been reviewed [71]. Alternative methods, such as the transcutaneous measurement of GFR using the fluorescent renal marker FITC-sinistrin [72,73], radiolabeled markers or unlabeled radiocontrast agents have been described in mice and canines [74], but were not used in the reported CKD–MBD studies [71,75,76]. Cystatin C is an endogenous marker frequently used in humans for GFR estimation and seems to be a sensitive marker in mice, but its use remains scarce in mice [71,77].

### 4.2. Further Biochemical Measurements

The number and the methods used to determine biological parameters were highly variable between studies, and findings, therefore, are difficult to compare. Blood calcium levels in CKD mice were similar [20,29,30,31,33,35,40,41,43,44,46,47,49,50,51,53,61,64,67], increased [19,21,28,37,38,48,56,58,60,63,65] or decreased [34,53,59,62] compared to control, depending on the studies and on the experimental conditions. 

Phosphatemia was either unaffected in CKD [21,28,31,33,37,38,41,42,43,44,47,48,49,51,53,60] or increased [19,20,27,29,31,35,40,43,46,50,53,56,58,59,61,62,63,64,65,67,68]. Surprisingly, serum phosphate levels were increased in some studies on mice fed an HP diet [44,54] but not in others [37,38], and the overall relation to serum urea and PTH levels is difficult to discern.

Serum PTH levels showed large variations. Mouse models with severe HPT were expected to present with a high turnover bone disease, but due to the plethora of different models, an array of different bone findings have been described. An increased PTH was found in some low-turnover bone disease models [55,63], and LDRL^−/−^ mice fed with a high-fat/cholesterol diet are described as resistant to the bone remodeling effect of SHPT [29,41]. While some authors reported no significant increase in PTH [28,30,31,33,43,66], it was mostly increased by 1.8- to 1000-fold compared to control mice [20,21,27,29,30,31,32,34,35,36,37,38,40,41,42,43,44,45,47,49,50,51,52,53,54,55,56,57,58,59,61,62,63,64,65,66,67,68]. PTH levels were increased in mice with CKD fed with a HP diet compared to mice fed with non-enriched diet [37,44,54]. In addition, the increase in PTH levels correlates with CKD severity [31,43,46,62].

FGF23 serum levels were increased by 3- to 1000-fold in CKD mice compared to control [27,30,31,34,36,43,46,47,50,51,53,54,58,59,60,61,62,63,64,65,66,68], alkaline phosphatase activity was unchanged or mildly increased [34,37,38,49,53,54,57,65], whereas plasma calcitriol was unchanged [61], decreased [47,49,50,51,59,61,62] or increased [46,58]. While comparing studies with different designs and heterogeneous genetic backgrounds, there is no evidence for a correlation between bone turnover and abnormal calcium/phosphate/PTH homeostasis. Biochemical markers of bone resorption and formation were also used in a few studies with different biomarkers and different designs but will not be discussed here [46,57,65,67].

## 5. Bone Analysis

### 5.1. Histomorphometry

The gold standard technique to determine trabecular bone microarchitecture and bone remodeling dynamics remains bone histomorphometry, performed ex vivo in mice. Different from humans, where a small iliac crest biopsy is available, the analysis in a mouse’s whole bone can be performed after the sacrifice. Some specificities in rodents, such as difficulty in recognizing osteoclasts, exist, and specific embedding and staining have to be used [78]. Both human and mouse bone analysis by histomorphometry require specialists able to interpret the samples [12,79] and only allow a two-dimensional (2D) analysis that requires “calculations” to move into 3D.

The same nomenclature and classification as in humans were frequently used in rodent studies in the absence of a specific nomenclature for rodents. The current classification and treatment strategy for ROD in humans is based on changes in bone turnover, mineralization and volumes (Table 2) [6,13] and requires a double tetracycline administration prior to the biopsy of the iliac crest to obtain dynamic parameters [7,80]. Parfitt et al. described and developed a standardization and nomenclature for bone histology in 1985 that was updated in 2012 [79,81] and is still in use.

The turnover reflects the rate of skeletal remodeling resulting from the balance between bone resorption and formation and is assessed by double labeling and corresponding bone formation rate or activation frequency. Measurements of osteoblasts surface and number, osteoid surface, osteoclasts number and bone eroded surfaces are related to bone turnover and are indicative, even though they are less accurate than the double labeling. Bone turnover is affected by different parameters, including PTH.

Osteitis fibrosa is a high-turnover bone disease secondary to SHPT, and mineralization defects in mixed uremic osteodystrophy are most often attributed to vitamin D deficiency [2,7]. 

The mineralization reflects the amount of unmineralized osteoid and is assessed by static measurements, such as osteoid volume and thickness, and by dynamic parameters (e.g., mineralization lag time) [7].

Volume indicates the amount of bone per unit volume of tissue and is assessed by measurements of bone volume on cancellous bone. Cortical and cancellous bone volumes can be differently affected by CKD.

Bone volume is not classically used to stratify bone diseases. Bones with a low turnover and normal mineralization are classified as adynamic bone disease, whereas those with a high bone turnover, especially when they exhibit other signs of high PTH, have osteitis fibrosa. Osteomalacia is diagnosed in case of abnormal mineralization and low bone formation, whilst bones with abnormal mineralization and a low bone formation have mixed disease [7,82], as illustrated in Table 2.

The bones most frequently studied in mice are the proximal and distal femora [21,29,30,31,33,36,37,40,41,42,43,47,48,49,50,52,53,54,55,57,58,59,60,61,62,63,64,68] but also proximal and distal tibia [19,27,32,66], lumbar and thoracic vertebra [28,39], mandible [38,51] or both vertebra and long bone [44,46]. Results of bone analysis can differ in vertebra and long bone [35], and this is consistent with human data [83]. The presence of marrow fibrosa and cortical bone measurements were also frequently reported.

Some studies only give some qualitative analysis. The number of analyzed parameters was highly variable from one study to another, and in Table 3, we report the most frequently used. Some studies analyzed one or more formation parameters, such as osteoid surface, volume and osteoblast quantification. Osteoid volume and surface were frequently increased in CKD. Other also analyzed resorption parameters such as osteoclasts quantification and eroded surface, and an increase of the latter was often observed. Trap staining can be used to quantify the osteoclasts [39,52]. Structure parameters such as bone volume/trabecular volume, trabecular thickness, numbers or spacing were frequently reported, and these parameters were variably affected by CKD.

Tetracycline-derived labels are the gold standard in humans to describe bone turnover. Double labeling was also performed in mice with various protocols and various parameters. However, differences in the formation parameters assessed by double labeling were not always significant, and authors used either formation or resorption parameters to classify into a high or a low turnover disease. In only a few studies, dynamic parameters in histomorphometry analysis were not considered [19,37,38,39,47,49,51,54,59,68]. In contrast, one or more dynamic parameters, such as mineral apposition rate, mineralization lag time, adjusted apposition rate and bone formation rate assessed thanks to the labeling, were performed in the majority of the studies with intraperitoneal or subcutaneous injections of fluorochromes [21,27,28,29,30,31,32,33,36,40,41,43,44,46,48,52,53,55,57,58,60,61,63,64,66] (Table 3). The time of the first fluorochrome injection (4 to 14 days before sacrifice), the second injection (2 to 3), as well as the delay between injections, vary (2 to 5 days). One study used triple labeling with injections 5 weeks, 2 weeks and 2 days before necropsy [66]. The main fluorochromes used were calcein, tetracyclin, alizarin, demeclocycline and xylene orange with various combinations in the studies.

The expression of specific markers from bone cell subtypes (for example, klotho, FGF23, RANKL and OPG, TNAP, osteocalcin, Phospho1) was frequently determined by immunohistochemical staining and quantitative PCR [27,33,39,46,57,58,59,65,68,84]. These analyses provide insight into the molecular machinery of bone and into the regulation of systemically active hormones such as FGF23, secreted by osteocytes and regulated by osteocyte protein dentin matrix protein 1, which is downregulated in CKD [61].

### 5.2. High-Resolution Imaging

Bone microarchitecture abnormalities can be assessed by high-resolution radiology, e.g., two-dimensional (2D) imaging with resolutions of 200 μm or two-photon absorptiometry (DXA). Three-dimensional HR-p-QCT imaging allows accessing the trabecular microarchitecture with an isotropic resolution of 82 μm [85,86,87,88]. 

Images of whole mouse bones can be obtained by micro-CT, from X-rays or more sophisticated from a synchrotron [85,86]. Micro-CT allows for a higher spatial resolution (6 to 20 μm) and the analysis of trabecular microarchitecture in three dimensions and informs on the cortical structure. These results showed good correlations with histological findings in animals and humans [89,90,91]. Specific micro-CT analysis guidelines were described for rodents [92].

In many studies, micro-CT or p-QCT were used in combination with histomorphometric analyses [10,27,28,30,31,33,34,36,37,38,39,43,44,45,46,50,51,52,54,56,57,58,61,63,65,66,68], but a few studies only relied on bone imaging [20,35,62,67]. The bones used for micro-CT or p-QCT analysis were proximal or distal femora, proximal or distal tibia, lumbar vertebra, thoracic vertebra and mandible. The type of bone used for micro-CT analysis sometimes differed from that used for histomorphometry [27,33,50,58,85]. Cortical and trabecular parameters were generally assessed, but some authors only described cortical [53,61,66] or trabecular parameters [34,39,58,68]. Some studies performed micro-CT in vivo for a follow-up [35,53,54]. Note that cortical alterations were frequently found in CKD mice [20,21,27,28,30,31,36,37,38,42,43,44,45,46,51,52,53,54,56,57,61,63,65,66,67]. As observed by histomorphometry, trabecular parameters were variably affected by CKD. The main cortical and trabecular parameters used are summarized in Table 4. Parameters analyzed by micro-CT and histology were sometimes different, but these studies were not designed for such a comparison, and the bone area and structure were often different from the area studied histologically [21,28,36,44,58,63].

### 5.3. Other Bone Exploration Methods Used

We recently proposed the use of in vivo quantitative bone planar scintigraphy to investigate bone remodeling in mice with or without CKD [44,93]. We designed a quantitative evaluation of bone uptake of phosphonate tracer on the knee’s regions of interest (ROI) at the epiphyseal plate regions, drawn on bone planar scintigraphic images, as a measure of osteoblast activity. An index was calculated from the counts in the knee’s ROI (normalized by pixels and seconds), corrected for activity administered, decay between administration and imaging and individual animal weights. This index was applied in healthy and CKD mice.

Mechanical properties were frequently studied by nanoindentation analysis via micromechanical anisotropy or by biomechanical three- or four-point bending tests [33,34,35,45,52,56,57,58,66]. Standard X-ray was used for implant examination [49,50]. FTIR microspectroscopy was used to determine chemical parameters, such as mineral-to-matrix ratio and mineral or collagen maturity [35].

Titanium implant resistance was evaluated by a biomechanical push-in resistance test and by an evaluation of bone-implant contact [47,49,50]. Bone calcium content was quantified by ash studies [33,34,62], and pyrophosphate content by fluorimetric pyrophosphate assay [27].

## 6. Mouse Models of CKD–MBD with Low and High Turnover ROD

The high bone turnover disease is the most frequently described phenotype in CKD–MBD mice [20,21,27,28,32,33,34,35,36,37,38,39,42,43,44,45,46,52,53,54,56,57,58,59,60,61,64,65,67,68]. Low bone turnover, either adynamic bone disorder or osteomalacia, was also described under specific experimental conditions [29,30,31,40,41,55,63,66] (Table 1): in surgical models, Lund et al. used C57BL/6 mice and administrated a low-phosphate together with calcitriol supplementation to generate mice with the adynamic bone disorder [40]. Frauscher et al. transiently fed DBA/2 mice with a high-phosphate diet, who developed phosphate nephropathy, CKD and low turnover osteodystrophy, despite high serum PTH concentrations [55]. Others used LDRL^−/−^ mice fed with high-fat/cholesterol diets [29,30,31,41] to induce low bone turnover disease. Mice fed with adenine for 6 weeks exhibited a decreased bone formation rate, i.e., a low bone turnover disease, possibly due to the prolongated adenine diet [66]. In contrast, several other studies with prolongated adenine diet induced a high turnover phenotype [46,52,53,67]. A remarkable mouse model of CKD has been established that circumvents systemic toxicity and surgical interventions and mimics progressive glomerular disease by the generation of an inducible podocyte-specific ablation of an essential endogenous molecule, the chromatin structure regulator CCCTC-binding factor (CTCF), which leads to rapid podocyte loss in iCTCFpod^−/−^ mice. These mice develop severe progressive albuminuria, hyperlipidemia, hypoalbuminemia and impairment of renal function and die within 8–10 weeks [55]. These mice develop high serum phosphate, PTH and FGF23 concentrations, together with bone mineralization defects, increased bone resorption and bone loss. NG et al. established an alternative mouse model of low turnover bone disease independent of CKD based on genetic osteoblast ablation and pamidronate treatment to inhibit osteoclastic activity [94].

## 7. Conclusions

In conclusion, an array of CKD–MBD mouse models has been developed over the past 20 years. These models recapitulate the bone phenotypic spectrum of ROD as present in humans with CKD, and pathophysiological mechanisms are largely concordant. Due to the complex network of underlying pathomechanisms of MBD in CKD and the various phenotypes of ROD, it is, however, still challenging to choose the most appropriate mouse model to address a specific scientific question. Research teams have to consider the strengths and limitations of each mouse model carefully. Due to the great heterogeneity in the protocols and the parameters studied, careful interpretation of the findings is required. On the other hand, experimental mouse models allow controlling, and thus avoiding confounding factors, to provide valuable insights into specific pathomechanisms of CKD–MBD and the therapeutic potential of respective interventions.

## Figures and Tables

**Table 1:** Summary of non-genetic mouse models of CKD–MBD.

(**a**) Summary of non-genetic mouse models of CKD–MBD.								
**Previous Reports**	**CKD Mice Model and Intervention**	**Genetic Strain**	**Number** **Gender,** **Age at CKD**	**Time Interval between CKD Induction and Bone Analysis**		**Diet**	**ROD**	**Objective of the Study**
Gagnon et al. [19]	Surgery	C57BL/6	87 F, 7 weeks	6 weeks		Standard	Osteitis fibrosa, high turnover	CKD model description
Matsumoto et al. [20]	Surgery	NA	16 M 6 weeks	20 weeks		HP	Osteitis fibrosa, high turnover	Relation of aortic mineral elements and ROD
Gonzalez et al. [32]	Surgery and BMP-7 treatment ±CaCO_3_	C57BL/6	33 M, 7 weeks	3 to 6 weeks		Standard	Osteitis fibrosa, high turnover	Impact of exogenous BMP-7
Heveran et al. [33]	Surgery	C57BL/6	26 M 11 weeks	11 weeks		Standard	Osteitis fibrosa, high turnover	Bone quality in CKD
Hou et al. [34]	Surgery	C57BL/6	36 M 9 weeks	12 weeks		HCD	Osteitis fibrosa, high turnover	Role of calcium supplementation on ROD
Kadokawa et al. [35]	Surgery	Crlj:CD1	16 M 7 weeks	3 to 19 weeks		Standard, HP	Osteitis fibrosa, high turnover	Mechanical properties, age relation
Lau et al. [37]	Surgery	DBA/2	21 F 20 weeks	12 weeks		Standard, HP	Osteitis fibrosa, high turnover	Effect of normal and HP diet in CKD–MBD
Lee et al. [38]	Surgery	DBA/2	n > 16 20 weeks	13 weeks		Standard, HP	Osteitis fibrosa, high turnover	Mandible in CKD mice with HP and standard diet
Li et al. [39]	Surgery and Osthole treatment	C57BL/6	26 10 weeks	8 weeks		Standard	Osteitis fibrosa, high turnover	Effect of osthole in ROD
Lund et al. [40]	Surgery, Calcitriol and/or BMP-7 treatment	C57BL/6	50 M, 14 weeks	12 weeks		Standard, low phosphate	Adynamic bone disorder, low turnover	Role of exogenous BMP-7
Zheng et al. [45]	Surgery and cinacalcet treatment	C57BL/6	18 M 6 to 8 weeks	8 weeks		Standard	Osteitis fibrosa, high turnover	Effects of cinacalcet on ROD
Zhang et al. [47]	Surgery, Titanium implants, Ovariectomy	C57BL/6	40 9 weeks	12 weeks		Standard	Unknown	Titanium implant in CKD and estrogen deficiency
Liu et al. [49]	Surgery, Titanium implants and vitamin D treatment	C57BL/6	30 F 10 weeks	13 weeks		Standard	Unknown	Role of vitamin D in fixation of titanium implants
Sun et al. [50]	Surgery, Titanium implants and FGF23 antibody	C57BL/6	32 F 10 weeks	12 weeks		Standard	Unknown	Implant osseointegration in CKD mice with FGF23 neutralization
Guo et al. [51]	Surgery Ovariectomy	C57BL/6	40 F 11 weeks	12 weeks		Standard	Unknown	Role of estrogens on mandible in CKD
Metzger et al. [52]	Adenine treatment	C57BL/6	32 M/F 16 weeks	10 weeks		Standard	Osteitis fibrosa, high turnover	Evaluation of gender effect in CKD-related ROD
Tani et al. [53]	Adenine treatment and TNAP inhibitor treatment	C57BL/6	38 M 8 weeks	12 weeks		HP	Osteitis fibrosa, high turnover	Effects of TNAP inhibitor in CKD–MBD
Tani et al. [54]	Adenine treatment	C57BL/6	35 M 9 weeks	12 weeks		Standard or HP 2, 4, 6 weeks	Osteitis fibrosa, high turnover	Description of a new CKD–MBD model and role of HP diet
Frauscher et al. [55]	HP diet	DBA/2	8 F 8 weeks	12 weeks		HP 4 and 7 days	Low turnover	Description of a new CKD–MBD model and role of HP diet
Chiu et al. [56]	Adenine diet and cinacalcet treatment	C57BL/6	20 M 8 weeks	6 weeks		HP	Osteitis fibrosa, high turnover	Effect of cinacalcet on ROD
Abbreviations: CKD, chronic kidney disease; CKD–MBD, chronic kidney disease–mineral bone disorder F, female, M, male; NA, not available; TNAP, tissue-nonspecific alkaline phosphatase; HP, high phosphate; HCD, high calcium diet; ROD, renal osteodystrophy; duration of CKD in the surgical model starts after the second surgery.								
(**b**) Summary of genetic mouse models of CKD–MBD.								
**Previous Reports**	**CKD Mice Model and Intervention**	**Genetic Strain**	**Number** **Gender, Age at CKD**	**Genetic Background**	**Time Interval between CKD Induction and Bone Analysis**	**Diet**	**ROD**	**Objective of the Study**
Liu et al. [57]	PKD model and TGF β antibody treatment	C57BL/6	>30 M	Jck mice	12 or 16 weeks of age	HP	Osteitis fibrosa, high turnover	TGF β Neutralization effect
Sabbagh et al. [58]	PKD model	C57BL/6	>40 F	jck mice	6 to 15 weeks of age	HP	Osteitis fibrosa, high turnover	Temporal biochemical and morphometric changes in PKD model
Stubbs et al. [59]	Autosomal recessive Alport model	NA	>40 F/M	Col4a3^−/−^FGF23^þ/eGFP^	8 to 14 weeks of age	Standard	Osteitis fibrosa, high turnover	Temporal changes of FGF23
Williams et al. [60]	X-linked Alport model and ligand trap of the RAPIIA treatment	C57BL/6	46 M	Col4a5^y/−^	28.5 weeks of age	Standard	Osteitis fibrosa, high turnover	Effect of ligand trap of the RAP IIA
Dussold et al. [61]	Autosomal recessive Alport model and DMP1 treatment	C57BL/6	>48 M	Col4a3^−/−^DMP1^TG^	8 and 23 weeks of age	Standard	Osteitis fibrosa, high turnover	Role of DMP1 using genetic and therapeutic approaches
Zhang et al. [62]	Autosomal recessive Alport model	NA	>96 F/M	Col4a3^−/−^	8 and 12 weeks of age	Standard/low phosphate/phosphate deficient	Unknown	Impact of phosphate restriction on FGF23 metabolism
Christov et al. [63]	Podocytopathy model	C57BL/6	n > 40 M/F 6 weeks	Pod^−/−^	8 weeks	Standard	Osteomalacia, low turnover	Description of a new CKD–MBD model with inducible podocyte-specific deletion
Abbreviations: CKD, chronic kidney disease; PKD, polycystic kidney disease; KO, knockout; RAPIIA, activin receptor type IIA; F, female, M, male; HFC, high fat/cholesterol; HF, high fat; NA, not available; HFC, high fat/cholesterol; HP, high phosphate; ROD, renal osteodystrophy.								
(**c**) Summary of mouse models of CKD with genetic modifications.								
**Previous Reports**	**CKD Mice Model and Intervention**	**Genetic Strain**	**Number** **Gender, Age at CKD**	**Genetic Background**	**Time Interval between CKD Induction and Bone Analysis**	**Diet**	**ROD**	**Objective of the Study**
Nikolov et al. [21]	Surgery	C57BL/6	48 F 10 weeks	APO-E^−/−^	10 weeks	Standard	Osteitis fibrosa, high turnover	Evaluation of vascular and bone axis in APO-E^−/−^ mice
Andrukhova et al. [27]	Surgery	C57BL/6	38 M 13 weeks	FGF23^−/−^VDR^−/−^	8 weeks	HCD lactose	Osteitis fibrosa, high turnover	Role of FGF23
Cejka et al. [28]	Surgery	C57BL/6	73 F 14 weeks	SOST^−/−^	12 weeks	Standard	Moderately increased turnover	Role of Sclerostin
Davis et al [29]	Surgery, BMP-7 and CaCO_3_ treatment	C57BL/6	64 F/M 14 weeks	Ldlr^−/−^ *	14 weeks	Standard, HFC	Adynamic bone disorder, low turnover	Exogenous BMP-7 role in CKD and metabolic syndrome
Fang et al. [30]	Surgery and Dkk1 antibody	C57BL/6	>40 14 weeks	Ldlr^−/−^ *	8 weeks	HF	Adynamic bone disorder, low turnover	Effect of neutralization of DKK1 in early CKD
Fang et al. [31]	Surgery	C57BL/6	>40 14 weeks	Ldlr^−/−^ *	8 and 14 weeks	HF	Adynamic bone disorder, low turnover	Description of CKD–MBD physiopathology in early CKD
Kaesler et al. [36]	Surgery	C57BL/6	n > 34 F 36 to 38 weeks	SOST^−/−^	12 weeks	HP	Osteitis fibrosa, high turnover	Role of Sclerostin
Mathew et al. [41]	Surgery, Sevelamer treatment	C57BL/6	n > 60 F/M 12 weeks	Ldlr^−/−^ *	16 weeks	Standard, HFC	Adynamic bone disorder, low turnover	Sevelamer effect in CKD and metabolic syndrome
Nikolov et al. [42]	Surgery, lanthanum and sevelamer treatment	C57BL/6	48 F 10 weeks	APO-E^−/−^	10 weeks	Standard	Osteitis fibrosa, high turnover	Sevelamer and Lanthanum effect in CKD and metabolic syndrome
Sugatani et al. [43]	Surgery, ligand trap of the RAPIIA treatment	C57BL/6	56 M 14 weeks	Ldlr^−/−^ *	14 weeks	HF	Low turnover /high turnover	Effect of ligand trap RAP type II A
Zaloszyc et al. [44]	Surgery	C57BL/6	>60 M 12 weeks	Gαq/11^−/−^	12 weeks	Standard HP	Osteitis fibrosa, high turnover	Role of specific osteoblast inactivation of PKC
Kaludjevoric et al. [46]	Surgery or adenine treatment	NA	80 F/M 7 weeks	Prx1-Cre; Klotho fl/fl	8 weeks	Standard	Osteitis fibrosa, high turnover	Role of klotho in CKD using klotho knockout in long bone mice
Barreto et al. [48]	Surgery, intraperitoneal pyrophosphate injection	NA	114 F 10weeks	APO-E^−/−^	10 and 16 weeks	Standard	Osteitis fibrosa, high turnover	Effect of pyrophosphate
Schiavi et al. [64]	Adenine treatment, sevelamer treatment	NA	>48	Npt2b^−/−^	5 weeks	Standard, HP	Osteitis fibrosa, high turnover	Sevelamer effect in CKD and Npt2b^−/−^ model
Hsu et al. [65]	Adenine treatment	C57BL/6	24 M 5 weeks	Phospho1^−/−^	8 weeks	Standard	Osteitis fibrosa, high turnover	Role of PHOSPHO1
Gardinier et al. [66]	Adenine treatment	C57BL/6	20 M/F 8 weeks	PPR^cKO^	6 weeks	Standard	Low turnover	Role of PPR in CKD osteocytes
Tatsumoto et al. [67]	Adenine treatment and lithium chloride treatment	C57BL/6	24 M 8 weeks	GSK-3β^+/−^	6 weeks	Standard	Osteitis fibrosa, high turnover	Effects of GSK-3βinhibition by genetic and treatment
Lin et al. [68]	Adenine treatment, Klotho knockdown via siRN, TSA treatment	C57BL/6	24 M	Klotho^−/−^	6 weeks	Standard	Osteitis fibrosa Osteitis fibrosa, high turnover	Role of Klotho loss and therapeutic effect of klotho restoration via TSA
Abbreviations: CKD, chronic kidney disease; KO, knockout; F, female, M, male; receptor; NA, not available; SOST, sclerostin; HFC, high fat/cholesterol; HP, high phosphate; HCD, high calcium diet; TSA, trichostatin A; fl, flox; ROD, renal osteodystrophy; PPR, PTH/P; H-related protein type 1 receptor; GSK, glycogen synthase kinase; RAPIIA, activin receptor type IIA; * Ldlr^−/−^ leads to a mild form of CK; the model is associated with surgery to induce more severe CKD.								

**Table 2:** Classification of renal osteodystrophy based on turnover and mineralization. The bone volume can be low, normal or high in the various forms of ROD.

Type of Renal Osteodystrophy	Histomorphometric Description
Osteitis fibrosa	Increased turnover, normal mineralization
Osteomalacia	Decreased turnover, abnormal mineralization
Adynamic bone disorder	Decreased turnover, normal mineralization (decreased cellularity)
Mixed osteopathy	Increased turnover, abnormal mineralization

**Table 3:** Histomorphometric findings based on the articles reviewed and presented in Table 1. Only findings of mice with CKD on normal and high phosphate diets are depicted, but no findings from specific intervention groups.

	Parameter	Abbreviation	Main Finding (Number of Articles)
Structural parameters	Cortex width	Ct. Wth	unmodified (1)
	Bone volume per Total Volume	BV/TV	unmodified (13), increased (7), decreased (6)
	Trabecular thickness	Tb.Th	unmodified (9), increased (3), decreased (2)
	Trabecular number	Tb.N	unmodified (9), increased (5), decreased (1)
	Trabecular spacing	Tb.Sp	unmodified (8), decreased (4)
	Bone surface area	BS/TV	unmodified (1)
	Wall thickness	WTh	unmodified (1), decreased (1)
Remodeling static parameters resorption	Osteoclast surface	Oc.S/BS	Increased (9), unmodified (7)
	Osteoclast number	Oc.N	unmodified (1), increased (1)
	Osteoclast number per Bone perimeter or surface	N.Oc./B.Pm or BS	unmodified (7), increased (6)
	Osteoclast volume density	NOc/T.Ar	increased (2), unmodified (1)
	Eroded surface	ES/BS	Increased (6), unmodified (6), decreased (1)
Remodeling static parameters formation	Osteoid volume	OV/BV	increased (8), unmodified (7), decreased (2)
	Osteoid thickness	O.Th	unmodified (4), increased (4)
	Osteoid surface	OS/BS	increased (8), unmodified (3)
	Osteoblast density	NOb/T.Ar	increased (2), unmodified (1)
	Osteoblast number	Ob.N	unmodified (3), increased (2), decreased (1)
	Osteoblast perimeter or surface density	Nob/BPm	increased (3), unmodified (3), decreased (1)
	Osteoblast surface	Ob.S/BS	unmodified (5), increased (4), decreased (1)
Remodeling dynamic parameters	Mineralizing surfaces per bone surface	MS/BS	unmodified (7), decreased (3), increased (1)
	Mineralizing surfaces per Osteoid Surface	MS/OS	decreased (4), unmodified (2)
	Single-labeled surface	sLS/BS	unmodified (2)
	Double-labeled surface	dLS/BS	unmodified (2)
	Mineral apposition rate	MAR	unmodified (11), increased (5), decreased (2)
	Bone formation rate	BFR/BS	unmodified (12), decreased (5), increased (1)
	Adjusted apposition rate	Aj.Ar	increased (4), unmodified (3), decreased (1)
	Mineralization Lag Time	MLT (day)	unmodified (5), increased (3), decreased (1)

**Table 4:** Micro-CT and P-QCT findings based on the articles reviewed and listed in Table 1. Only findings of mice with CKD on normal and high phosphate diets are depicted, but no findings from specific intervention groups.

Cortical Parameters	Abbreviations	Main Finding
Average cortical thickness	Ct.Th	decreased (13), unmodified (5)
Cortical bone volume per Total volume	Ct.BV/TV	decreased (6), unmodified (1)
Cortical mean bone mineral density	Ct BMD	decreased (9), unmodified (3)
Total volume	TV	decreased (1)
Total cross-sectional area inside the periostal envelope	Tt.Ar	unmodified (3), increased (1), decreased (1)
Marrow area	Ma.Ar	unmodified (1)
Bone area fraction	Ct.Ar/Tt.Ar	increased (2), unmodified (1)
Cortical bone area	Ct.Ar	decreased (4), unmodified (3), increased (1)
Cortical Bone Porosity	Ct.por	increased (6), unmodified (1)
Trabecular parameters	abbreviations	Main finding
Mean bone mineral density	BMD or TMD (total or trabecular)	decreased (9), unmodified (3), increased (1)
Bone volume per Total volume	BV/TV	decreased (11), unmodified (7), increased (5)
Trabecular thickness	Tb.Th	unmodified (10), decreased (7), increased (1)
Trabecular number	Tb.N	unmodified (9), decreased (8), increased (3)
Trabecular spacing	Tb.Sp	unmodified (9), increased (8), decreased (1)
Connectivity density	Conn.D	unmodified (3), decreased (3)
Structure model index	SMI	decreased (3), increased (1), unmodified (1)
Anisotropy	DA	unmodified (2)

## Data Availability

No new data were created.
